# HIV Drug Resistance Early Warning Indicators in Namibia for Public Health Action

**DOI:** 10.1371/journal.pone.0065653

**Published:** 2013-06-07

**Authors:** Anna Jonas, Justice Gweshe, Milner Siboleka, Michael DeKlerk, Michael Gawanab, Alfons Badi, Victor Sumbi, Dawn Pereko, Abraham Blom, Samson Mwinga, Michael R. Jordan, Logan Jerger, Kiger Lau, Steven Y. Hong

**Affiliations:** 1 Directorate of Special Programmes, Republic of Namibia Ministry of Health and Social Services, Windhoek, Namibia; 2 Management Sciences for Health, Windhoek, Namibia; 3 Strengthening Health Outcomes through the Private Sector, Abt Associate Inc, Windhoek, Namibia; 4 Department of Public Health and Community Medicine, Tufts University School of Medicine, Boston, Massachusetts, United States of America; 5 Division of Geographic Medicine and Infectious Diseases, Tufts Medical Center, Boston, Massachusetts, United States of America; Alberta Provincial Laboratory for Public Health/University of Alberta, Canada

## Abstract

**Background:**

HIV drug resistance (HIVDR) testing is not routinely available in many resource-limited settings, therefore antiretroviral therapy (ART) program and site factors known to be associated with emergence of HIVDR should be monitored to optimize the quality of patient care and minimize the emergence of preventable HIVDR.

**Methods:**

In 2010, Namibia selected five World Health Organization Early Warning Indicators (EWIs) and scaled-up monitoring from 9 to 33 ART sites: *ART prescribing practices, Patients lost to follow-up (LTFU) at 12 months, Patients switched to a second-line regimen at 12 months, On-time antiretroviral (ARV) drug pick-up,* and *ARV drug-supply continuity.*

**Results:**

Records allowed reporting on three of the five selected EWIs. 22 of 33 (67%) sites met the target of 100% initiated on appropriate first-line regimens. 17 of 33 (52%) sites met the target of ≤20% LTFU. 15 of 33 (45%) sites met the target of 0% switched to a second-line regimen.

**Conclusions:**

EWI monitoring directly resulted in public health action which will optimize the quality of care, specifically the strengthening of ART record systems, engagement of ART sites, and operational research for improved adherence assessment and ART patient defaulter tracing.

## Introduction

As of December 2011, over 8 million people infected with HIV were receiving antiretroviral therapy (ART) in low- and middle-income countries which represents a 26-fold increase since 2003 [1]. Due to HIV’s error-prone replication, high mutation rate and viral recombination, development of some HIV drug resistance (HIVDR) is inevitable, even with appropriate ART prescribing and adherence [2]–[3]. HIVDR has significant human and financial implications: it limits treatment options; second-line ART regimens involve more long-term toxicity; and annual costs of second-line regimens are 4–8 times higher than that of currently recommended first-line regimens [4]. As the number of people on treatment increases, the emergence of meaningful population-level HIVDR becomes a greater risk which has the potential to undermine the dramatic gains that ART programs have had in reducing the morbidity and mortality of HIV-infected people in resource-limited settings [5].

Monitoring of ART program factors known to be associated with the emergence of HIVDR for the purpose of improving programmatic functioning, may minimize the emergence of preventable HIVDR, especially at ART sites where viral load and HIVDR testing is not routinely available. For example: HIVDR testing is not required to predict the emergence of drug-resistant HIV in settings where inappropriate prescribing practices (mono- or dual-therapy), treatment interruptions due to suboptimal patient adherence, poor patient retention on ART, or ART supply shortages or stock-outs occur at unacceptably high levels. These factors have been shown to be associated with the development of HIVDR [6]–[12]; thus, their monitoring may alert national ART program planners to issues which may be adjusted to minimize the emergence of HIVDR.

### HIVDR Early Warning Indicators

The foundation of the World Health Organization’s (WHO) global HIVDR prevention and assessment strategy [13], which includes laboratory-based surveys of acquired [14] and transmitted [15] HIVDR, is the monitoring of HIVDR Early Warning Indicators (EWI). EWIs assess ART site and program factors potentially associated with HIVDR [16]. Utilizing data routinely collected in patients’ medical and pharmacy records, EWI monitoring is a minimum-resource strategy designed to be integrated into national monitoring and evaluation programs. EWIs survey factors related to patient care, patient behavior, and clinic-level and program management, all of which are associated with the emergence of HIVDR. When monitored annually at all or a large number of representative ART sites, EWIs provide countries with evidence to make programmatic adjustments at the level of an individual site or the country, when necessary. WHO updated their EWI definitions in 2012 [17].

### HIV in Namibia

Namibia is a resource-limited country in sub-Saharan Africa that has been severely affected by the HIV epidemic. In Namibia, there are approximately 200,000 people living with HIV in a population of 2.1 million [18]. Among 15–49 year olds, approximately 18.2% are infected with HIV-1 [19]. The epidemic is predominantly spread via heterosexual contact, and prevalence estimates vary by region with up to 37.7% infected with HIV-1 in the most heavily-affected areas in the north [19].

### ART rollout

ART has been available in Namibia’s private sector since 1997 and in the public sector since 2003. At 90%, Namibia has one of the highest ART coverage rates in Sub-Saharan Africa with 88,717 eligible patients on ART as of December 2010 [20]. At present, ART is available at all 40 public hospitals and at an additional 111 satellite/outreach service points, as well as 30 Integrated Management of Adolescent and Adult Illness (IMAI) sites [21]. Of these sites, the national ART program considers 35 to be main public ART sites. Main public ART sites are sites that dispense ART independently of other ART sites and dispense ART to patients at IMAI and satellite/outreach sites.

In the public sector, ART is provided free of charge following a population-based model of care with one primary first-line regimen and three alternate first-line regimens consisting of two nucleoside reverse transcriptase inhibitors (NRTI) combined with a non-nucleoside reverse transcriptase inhibitor (NNRTI). The recommended second-line regimen consists of 2 NRTIs with a ritonavir-boosted protease inhibitor (PI). ART initiation is based on WHO clinical staging and/or CD4 cell count ≤350 cells/mm^3^. All public ART sites have access to first and second-line ART regimens. At all public ART sites, viral load testing is performed six months after ART initiation and targeted viral load testing is performed to confirm clinical or immunological failure [21]. With support from Management Sciences for Health (MSH) Namibia, a standardized pharmacy record system, the Electronic Dispensing Tool (EDT), is used to dispense all ART. In the private sector, ART is provided utilizing an individual model of care with ART regimens selected based on results of drug resistance testing.

### Minimizing HIVDR

The Namibia Ministry of Health and Social Services (MoHSS) has been proactive in minimizing preventable HIVDR. Together with its national and international partners, the MoHSS publishes annual reports and national plans, following WHO recommendations, for the prevention and assessment of HIVDR. The national ART program mandates the use of standardized national ART prescribing practices, WHO pre-qualified drugs, and standardized medical and pharmacy record-keeping systems for national surveillance [21]. In 2009, Namibia piloted EWI monitoring in nine sites, which led to adjustments in the existing national data capture tools, as well as feedback and trainings for staff at each individual site [22]. The 2010 EWI exercise scaled up the monitoring efforts to all main ART sites.

## Methods

### Early Warning Indicators Selection

Namibia chose the following five indicators based on relevance to anticipated program interventions and availability of data: *ART Prescribing Practices*, *Patients lost to follow-up 12 months after ART initiation*, *Patients switched to second-line ART during first 12 months*, *On-time ARV drug pick-up*, and *ARV drug supply continuity* [16]. Definitions (numerator/denominator) for these selected EWIs and their respective recommended targets are summarized in Table 1. EWI definitions were based on previous (2010) WHO-EWI guidance [16] and not on the latest (2012) WHO EWI guidance [17].

**Table 1:** Selected 2010 WHO Early Warning Indicator definitions (Numerator/Denominator) and targets.

Indicator	Definition (Numerator/Denominator)
ART prescribing practices	Numerator: Number of adult patients initiating ART at the site who initially pick up from the pharmacy, an appropriate first-line ART regimen.*
	Denominator: Number of adult patients initiating ART at the site on or after the designated EWI sample start date.† Sampling continues until the full sample size is reached.
	Target: 100%
Patients lost to follow-up at 12 months#	Numerator: Number of adult patients initiating ART at the site who are lost to follow-up§ during the 12 months after starting ART.
	Denominator: Number of adult patients initiating ART at the site on or after the designated EWI sample start date. Sampling continues until the full sample size is reached.
	Target: ≤20%
Patients switched to a second-line regimen at12 months#	Numerator: Number of adult patients initiating ART at the site whose initial ART regimen was changed to a regimen that includes a different drug class during the first 12 months after ART initiation (including switches for regimen failure and substitutions for toxicity).
	Denominator: Number of patients initiating ART at the site on or after the designated EWI sample start date who are retained on ART 12 months after start. Sampling continues until the full sample size is reached.∞
	Target: 0%
On-time ARV drug pick-up#	Numerator: Number of adult patients who have picked up all their prescribed ARV drugs on time‡ for two consecutive drug pick-ups after a baseline pick-up.
	Denominator: Number of patients who picked up ARV drugs on or after the designated EWI sample start date. Sampling continues until the full sample size is reached.
	Target: ≥90%!
ARV drug-supply continuity	Numerator: Number of months in the designated year in which there were no stock-out∧ days of any adult ARV drug routinely used at the site.
	Denominator: 12 months.
	Target: 100%

ART - Antiretroviral therapy.

ARV - Antiretrovirals.

LTFU - Lost to follow-up.

EWI - Early Warning Indicator.

* Appropriate first-line ART regimen: An ART regimen that meets one or both of the following definitions:

• Standard regimen listed in national ART guidelines and used according to those guidelines.

• Regimen recommended in the WHO treatment guidelines.

† EWI sample start date: The date designated as the start of the sampling. The sample start date is fixed by the HIVDR Working Group.

§ Lost to follow-up: Patients who had not returned to the pharmacy or clinic ≤90 days after the last ART run-out date during the 12-months after the date of ART initiation were classified as LTFU. Transfers of care to another site and deaths were excluded from the numerator and denominator. Stopping therapy without restarting was classified as not LTFU if the patient continued to attend clinic appointments.

∞ Transfers of care to another site, deaths, and ART stop without a restart were excluded from the denominator.

‡ On-time pick-up of ARV drugs: A patient pick-up of ARV drugs on or before the date the previously dispensed drugs would have run out if they had been taken according to schedule.

! The following patients were excluded from the denominator: Patients who transferred out, died, or stopped ART without a restart between baseline pick-up date and baseline pick-up run-out date.

# For EWIs: *Patients LTFU at 12 month*, *Patients switched to a second-line regimen at 12 months*, and *On-time ARV drug pick-up,* if no ARV pick-up date, regimen, and number of pills dispensed was not recorded in the records, it was assumed that no pick-up had occurred, resulting in the most conservative estimate of each indicator.

∧Stock-out: Any occurrence of zero stock of a routinely-used ARV drug at the site at which the patient routinely picks up ARVs.

Table adapted from WHO HIV Drug Resistance EWI guidance document [16].

### Ethics Statement

Ethical review was not required as this data was public health surveillance data abstracted from existing routinely collected ministry of health medical records. Only anonymised data were abstracted from the medical records for public health surveillance purposes. Names, dates of birth, addresses, and unique patient identifier numbers were not abstracted from records. After discussion with the Tufts Medical Center institutional review board, it was determined that because this was routine public health de-identified data analyzed within the Ministry of Health and Social Services in Namibia, no formal written waiver was necessary. The data used for this study was obtained from and analyzed by the MoHSS of Namibia.

### Site Selection and Data Abstraction

All 35 main public ART delivery sites were selected for EWI abstraction in 2010, which included the nine sites that participated in EWI abstraction in 2009. Only main ART sites were selected for EWIs because satellite/outreach and IMAI sites are considered part of the main ART sites (utilizing same ART staff, pharmacists and record systems). Private ART sites were not selected for inclusion because they do not utilize a population-based model of care and have routine HIVDR testing available. Data abstraction was conducted in October 2010 centrally by a data abstraction team formed by the HIVDR technical working group (TWG) in collaboration with the WHO and the MoHSS. The team consisted of four members who had been trained on the WHO methodology of EWI data abstraction. Primary data were centrally queried from the EDT national database through automatic queries into an Excel tool provided by the WHO, which calculated results for each indicator.

### Data Quality Assessment

Data quality assessments were implemented throughout the EWI process. Three elements of data quality were considered in the assessments: data reliability, data completeness, and data consistency [16]. Data reliability, which is an assessment of the quality of the abstraction, was assessed by confirming 10% of the centrally-queried data to the existing data in the EDT. Data completeness was assessed from the centrally-queried data; and sites with a large percentage of data missing were removed from EWI analyses. Finally, assessment of data consistency was initially performed during the pilot of EWIs [22] and the most optimal source for each variable was determined. EDT data were considered the gold standard for pharmacy pick-up dates and ART regimens dispensed, while the national electronic patient management system (ePMS) and paper records (Patient Care Booklets) were considered gold standard for information about patient status such as dates of transfer in and transfer out, dates of death, and dates of stop. Therefore, EDT data for patients who had incomplete pill pick-ups were validated and corrected by comparing records in ePMS and Patient Care Booklets, looking for dates of transfer out, death or ART stop. Centrally-queried EDT data for patients who had inappropriate ART regimens at start or at 12 months were validated and corrected with the site-specific EDT system to ensure accuracy of the queries. Validation with ePMS and paper medical records was performed by the individual ART sites that were trained on EWI methodology at a national EWI conference.

### Sample Size

In order to make the results generalizable to the patient population at the ART site, the sampling strategy was based on calculating a minimum sample size for each indicator at each site, based on the number of eligible patients for each EWI. Two different cohorts of ‘eligible patients’ were formed: 1) Patients consecutively initiating ART for the first time on or after the EWI sample start date of July 1, 2008 (*ART prescribing practices*, *Patients LTFU at 12 month*, and *Patients switched to a second-line regimen at 12 months*), and 2) Patients consecutively picking up ART on or after the EWI sample start date of January 1, 2010, regardless of duration of regimen (*On-time ARV drug pick-up*). Sample size calculations were performed to provide a 95% CI of ±7%, assuming a true prevalence of 50%; this provided the most conservative estimate of the sample size required [16]. For ARV drug-supply continuity, data were abstracted on stock-outs of each ARV drug in routine use from January 1, 2009 until December 31, 2009 using a stock-take module in EDT, which records the stocks in the central store and dispensary at each site.

## Results

**Figure 1:**
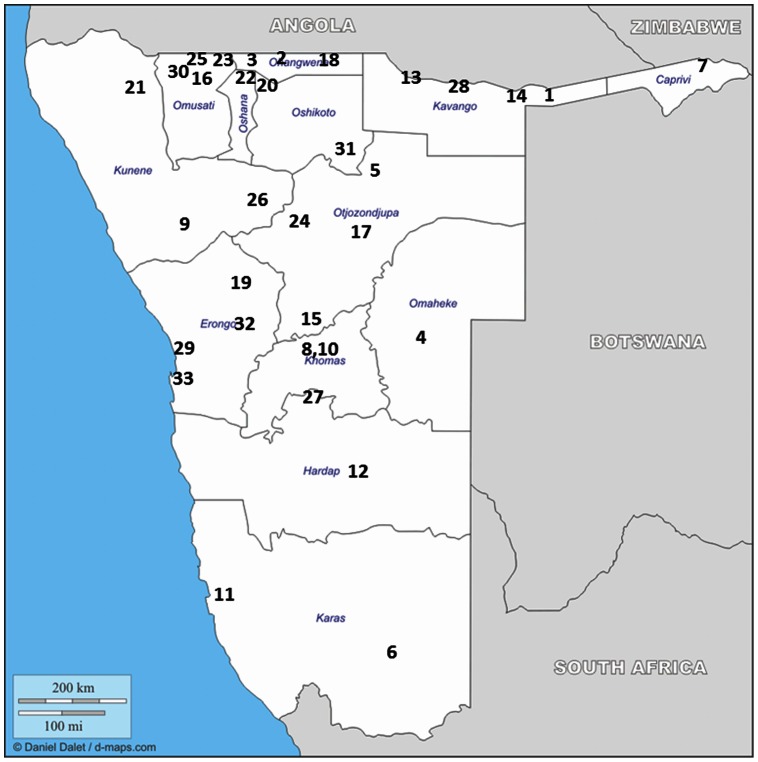
Geographic location of antiretroviral therapy sites. Adapted from original map available at: http://d-maps.com/carte.php?num_car=4824&lang=en.

Namibia abstracted data on three EWIs: *ART prescribing practices, Patients LTFU at 12 months,* and *Patients switched to a second-line regimen at 12 month* at 35 main ART sites (Figure 1). Two ART sites did not have complete data available so were removed from EWI analyses. Data from 3,875 patients were abstracted and analyzed. Site-specific EWI results are presented in Table 2, and the national EWI summary is presented in Table 3. *On-time ARV drug pick-up* and *ARV drug-supply continuity* could not be monitored because information entered into existing patient records was found to be incomplete or inaccurate. ART site profiles are presented in Table 4.

**Table 2:** Namibia site-specific EWI results.

ART site	Percent appropriate initial ART regimen prescriptions	Percent starting first-line ART lost to follow-up at 12 months	Percent starting first-line ARTwhose regimen was switched to second line during first 12 months
	(7/1/08–6/30/09)	(7/1/08–6/30/09)	(7/1/08–6/30/09)
	Target: 100%	Target: ≤20%	Target: 0%
	N (%)	N (%)	N (%)
1	89/89 (100)	**23/98 (23)**	0/93 (0)
2	120/120 (100)	16/111 (14)	0/119 (0)
3	74/74 (100)	**21/67 (31)**	0/72 (0)
4	127/127 (100)	**35/127 (28)**	**4/128 (3)**
5	**109/110 (99)**	**38/108 (35)**	**2/106 (2)**
6	22/22 (100)	**7/19 (37)**	0/19 (0)
7	153/153 (100)	**50/155 (32)**	**3/100 (3)**
8	**173/175 (99)**	**53/168 (32)**	0/150 (0)
9	**99/100 (99)**	17/92 (19)	**3/92 (3)**
10	144/144 (100)	**32/139 (24)**	**1/108 (1)**
11	120/120 (100)	**29/115 (25)**	**1/112 (1)**
12	98/98 (100)	9/95 (10)	0/93 (0)
13	100/100 (100)	13/97 (13)	**1/93 (1)**
14	**99/100 (99)**	**21/95 (22)**	**1/90 (1)**
15	**99/100 (99)**	13/98 (13)	0/99 (0)
16	133/133 (100)	9/119 (8)	0/122 (0)
17	61/61 (100)	**13/48 (27)**	**1/56 (2)**
18	100/100 (100)	3/95 (3)	0/95 (0)
19	**74/75 (99)**	9/69 (13)	0/74 (0)
20	180/180 (100)	7/175 (4)	0/82 (0)
21	100/100 (100)	6/99 (6)	0/94 (0)
22	**174/176 (99)**	**37/175 (21)**	**2/172 (1)**
23	155/155 (100)	19/148 (13)	**1/132 (1)**
24	**144/145 (99)**	20/134 (15)	**2/128 (2)**
25	160/160 (100)	17/159 (11)	0/92 (0)
26	100/100 (100)	**35/93 (38)**	**2/93 (2)**
27	**99/100 (99)**	18/99 (18)	**3/97 (3)**
28	175/175 (100)	30/175 (17)	**1/165 (1)**
29	**142/145 (98)**	**41/124 (33)**	**6/107 (6)**
30	110/110 (100)	7/107 (7)	**2/95 (2)**
31	**117/120 (98)**	**44/119 (37)**	**9/108 (8)**
32	42/42 (100)	**26/38 (68)**	0/30 (0)
33	154/154 (100)	13/150 (9)	0/133 (0)

Bold data indicates a site has not achieved the WHO-recommended target for that EWI.

ART - Antiretroviral therapy.

LTFU - Lost to follow-up.

EWI - Early Warning Indicator.

* Cohort of patients initiating ART at the site on or after the sample start date (July 1, 2008).

**Table 3:** National EWI summary.

EWI	EWI target for all sites (time period)	Number of sites meeting EWI target(% of sites meeting target)
Percent appropriate initial ART regimen prescriptions	Target: 100% (7/1/08–6/30/09)	22 of 33 (67%)
Percent starting first-line ART lost to follow-upat 12 months	Target: ≤20% (7/1/08–6/30/09)	17 of 33 (52%)
Percent starting first-line ART switched to second lineduring first 12 months	Target: 0% (7/1/08–6/30/09)	15 of 33 (45%)

ART - Antiretroviral therapy.

EWI - Early Warning Indicator.

**Table 4:** ART site profiles.

Site	Year ARTstarted	Geographical location (Region)	Patients on ART	Pre-ART patients	ART starters in 12 month	ART prescriber: patient ratio
1	2004	Kavango	821	1927	239	1∶205
2	2004	Ohangwena	3600	3157	489	1∶900
3	2004	Ohangwena	6157	4757	815	1∶1231
4	2004	Omaheke	1051	2149	396	1∶263
5	2004	Otjozondjupa	2115	91	408	1∶529
6	2004	Karas	685	164	152	1∶685
7	2004	Caprivi	5780	1894	1089	1∶723
8	2004	Khomas	3697	4498	1746	1∶616
9	2005	Kunene	328	142	115	1∶82
10	2003	Khomas	5500	6500	1800	1∶917
11	2004	Karas	2296	1154	450	1∶574
12	2005	Hardap	716	370	250	1∶179
13	2004	Kavango	1999	688	422	1∶666
14	2004	Kavango	1114	1731	941	1∶371
15	2005	Otjozondjupa	2000	NA	228	1∶1000
16	2004	Omusati	2247	1843	535	1∶562
17	2005	Otjozondjupa	338	49	86	1∶169
18	2004	Eenhana	1438	973	319	1∶360
19	2005	Erongo	700	528	156	1∶140
20	2000	Oshikoto	15971	3000	1897	1∶1597
21	2004	Kunene	851	589	200	1∶106
22	2003	Oshana	13967	4485	1746	1∶2327
23	2003	Omusati	2872	6709	792	1∶319
24	2004	Otjozondjupa	1577	1519	473	1∶1577
25	2003	Omusati	4534	4136	2000	1∶2267
26	2004	Kunene	567	814	279	1∶567
27	2003	Hardap	708	NA	290	1∶64
28	2003	Kavango	5052	2648	1120	1∶1684
29	2004	Erongo	2600	1417	445	1∶520
30	2004	Omusati	1326	922	1048	1∶1326
31	2004	Oshikoto	1487	1513	324	1∶1487
32	2005	Erongo	376	108	166	1∶376
33	2003	Erongo	4063	2559	945	1∶677

ART - Antiretroviral therapy.

NA - Data not available.

### ART Prescribing Practices

Twenty-two of 33 (67%) sites met the target of 100% appropriate initial ART regimen prescriptions, according to national ART guidelines [21]. (Table 2–3) Of the sites not meeting the target of 100%, all eleven sites achieved appropriate prescribing of 98–99%. No patient at any site was prescribed mono-or dual-therapy. Every patient who was prescribed an inappropriate first-line regimen was on an appropriate PI-based regimen. There were significant differences between pre-validated and validated data. Pre-validated data showed 8/33 (24%) sites met the target for *ART prescribing practices*. The validation process discovered that EDT automatic queries had included not only patients consecutively initiating ART for the first time, but some patients who were transferred in on ART from another site and patients who were on pre-exposure prophylaxis. EDT automatic queries also generated some incorrect starting regimens, which were revealed when validated with the dispensing record on EDT.

### Patients Lost to Follow-up at 12 Months

Seventeen of 33 (52%) sites met the target of ≤20% LTFU 12 months after ART initiation. (Table 2–3) The range of LTFU rates between ART sites was large from 3% to 68%. Pre-validated data showed 13/33 (42%) sites met the target for LTFU. The validation process discovered discrepancies between EDT and Patient Care Booklets, including differences in dates of death, dates of transfer out, patients in-transit, and ART start dates.

### Patients Switched to Second-line during first 12 Months

Fifteen of 33 (45%) sites met the target of 0% of ART initiators switched to a second-line regimen during the first 12 months. (Table 2–3) In sites not achieving the target of 0%, very few patients had been switched to second-line regimens (range 1%–8%). Pre-validated data showed 0/33 (0%) sites met the target of 0% switch to second line. The validation process revealed discrepancies between the automatic query for regimen at 12 months when compared with that in the EDT dispensing record.

### On-time ARV Drug Pick-up

Existing record keeping systems did not allow for monitoring of on-time pill pick-up at all ART sites. Following the 2009 EWI exercise, national record keeping tools were adapted to capture data necessary to monitor this EWI; however, these adjustments would not be expected to be available for the abstraction of these 2010 EWIs.

### ARV Drug-supply Continuity

Existing record keeping systems did not allow for monitoring of ARV drug stock-outs for all sites because drug stock-outs were not adequately recorded.

## Discussion

The purpose of implementing routine HIVDR EWI monitoring is to assess the extent to which ART programs are functioning to optimize prevention of HIVDR. EWI monitoring provides countries with an evidence base that can optimize programmatic functioning–both at the site and national level–thereby, leading to public health action to optimize patient care and minimize the emergence of preventable HIVDR. In 2010, Namibia implemented their second round of integrating EWI monitoring into routine national ART program functioning. With technical support from the WHO, Namibia adapted the WHO generic EWI abstraction guidance [16] with its existing human capacity and health systems infrastructure.

Based on clinical relevance and availability of data, Namibia selected five recommended WHO EWIs and scaled-up monitoring to 33 of 35 main ART sites throughout the country from the previous nine sites in 2009: *ART Prescribing Practices*, *Patients lost to follow-up 12 months after ART initiation*, *Patients switched to second-line ART during first 12 months*, *On-time ARV drug pick-up*, and *ARV drug supply continuity*. These 33 ART sites are meant to be representative of all public ART sites in the country. Overall, it was found that the existing pharmacy and medical record database (EDT, ePMS and Patient Care Booklets) allowed for valid monitoring of three EWIs: *ART Prescribing Practices*, *Patients lost to follow-up 12 months after ART initiation*, and *Patients switched to second-line ART during first 12 months*. Though the existing pharmacy and medical record database did not allow for adequate monitoring of the remaining two EWIs–*On-time ARV drug pick-up* and *ARV drug supply continuity*–the abstraction exercise itself served to inform the TWG about the limitations in the existing record-keeping system and give guidance on the necessary modifications needed for future EWI monitoring. The previous 2009 EWI pilot [22] resulted in modifications to the data capture tools such that all five EWIs could be abstracted. However, there was insufficient time between the 2009 EWI pilot and 2010 scale-up for the effects of the 2009 program implementations to be reflected in the scale-up results.

In 2008 to 2009, only 67% of ART sites met the WHO target of 100% for *ART Prescribing Practices*, which is somewhat lower than recently published data from other African settings (Bennett et al in 907 sites (74%) [4], Billong et al in 40 sites (90%) [23], Dzangare et al in 81 sites (88%) [24], and Sigaloff et al in 13 sites (85%) [25]). Importantly, although the percentage of sites meeting the target was low in Namibia, sites that did not meet the 100% target still had very few patients prescribed an inappropriate first-line regimen, and no patient were prescribed dual- or mono-therapy. All the inappropriate first-line regimens were in fact appropriate PI-based regimens which would not be expected to increase the risk of the emergence of HIVDR. Therefore, these data demonstrate overall successful implementation of national prescribing guidelines and training of ART staff in appropriate ART prescribing.

Only 52% of sites met the WHO target of ≤20% for *Patients lost to follow-up 12 months after ART initiation*, which is not unlike recently published data from other African countries (Bennett et al in 794 sites (59%) [4], Billong et al in 40 sites (20%) [23], Dzangare et al in 81 sites (50%) [24], and Sigaloff et al in 13 sites (92.3%) [25]). These data suggest that many patients are being lost to care within their first 12 months of ART and/or many patients are transferring out to alternate ART sites without informing their ART site. This patient population may be at high risk for experiencing ART treatment interruptions and developing HIVDR. The broad range of LTFU rates between sites suggests that there may be factors at the site-level that may be influencing a site’s ability to retain patients in care. These data have prompted the MoHSS to engage in public health action, specifically operational research to assess predictors of and reasons for LTFU and of intensifying defaulter tracing with the goal of re-engaging patients back into care within 48 hours of running out of pills. This intensification of defaulter tracing will include the following elements: 1) routinely updated patient locator information; 2) dedicated patient tracer; 3) generation of daily lists of defaulters; 4) daily tracing (phone and physical) of defaulters; and 5) standardized system of tracing deaths and transfers out. EWI results were also used in Papua New Guinea to strengthen their ART services by: 1) establishing a formalized referral system that documents patient transfers between clinics; and 2) regular review of patient clinic attendance and drug pickups to identify patients at risk for suboptimal adherence and ways to remove barriers to on-time pill pickup by providing subsidies for transportation and food for those in need [26].

Only 45% of sites achieved the target of 0% of *Patients switched to second-line ART during first 12 month*. However, in sites not meeting the target, very few patients were switched to second-line regimens during the first 12 months, suggesting appropriate physician prescribing practices and success in managing ARV toxicity and side effects through in-class substitutions. These data suggest that patients who were retained in care at 12 months had good clinical outcomes and were not failing therapy.

Similarly to previously reported EWI data in Namibia [22], data for *On-time ARV drug pick-up* were considered not to be a true reflection of population-level adherence in Namibia’s ART sites. In Namibia, routine pharmacy dispensing practice is meant to include the routine counting of remnant pills (number of pills left over from the previous prescription) and the dispensing of a specified number of days of pills. However, the number of remnant pills was not routinely recorded, thus it was not possible to calculate the actual pill run-out date, necessary to monitor this EWI. Instead, the importance of these results lie in the operational lessons learned which can be applied to the monitoring of the EWI in future years. Similar to trends in other countries monitoring EWIs, Namibia has begun to build capacity to monitor medicine possession ratio (MPR defined as numbers of days of pills dispensed/number of days in the interval), as MPR may be better than on-time ARV pickup for identifying patients at risk for HIVDR [25], [27]. MPR is not affected by variances in pill dispensing practices and next appointment dates by pharmacists, therefore, may allow for a more valid measurement of maximum ARV adherence. These data have prompted the MoHSS to engage in public health action, specifically operational research which showed MPR to be associated with short-term virologic suppression 6 months after starting ART in Namibia [28].

Similarly to previously reported EWI data in Namibia [22], it was not possible to assess *ARV drug supply continuity.* In the previous EWI pilot [22], existing pharmacy records did not capture stock at the level of the site dispensary but rather at a more central level which limited the country’s ability to assess drug stock at the dispensing point. Based on the pilot EWI exercise [22], programmatic changes were implemented to capture stock at the ARV dispensing point. However, pharmacists did not enter in stock into the electronic system in real-time and were allowed to dispense to patients on “0” stock. Therefore, it appeared in the record that many drug stock-outs occurred even when ARV drugs were available to the patients. Recognizing the importance of monitoring this EWI, as ARV drug stock-outs can be a cause of treatment interruptions and HIVDR [8]–[10], [12], Namibia is in process of applying these operational lessons learned to the monitoring of this important EWI in future years.

The successful 2010 EWI exercise and integration plan will provide Namibia a solid evidence base that can be used to make statements about national and site-specific programmatic functioning and potential HIVDR. This evidence base will serve to contextualize results from Namibia’s surveys of acquired HIVDR in patients starting ART and from surveys of transmitted HIVDR in specific geographic regions. The EWI data training and monitoring process has mobilized the national ART program and its partners to institute adjustments in existing databases, which will facilitate monitoring of WHO recommended EWIs in the future and which will yield a more accurate assessment of overall programmatic functioning. Additionally, EWI monitoring has prompted the national program in further public health action, specifically engaging sites in dialogue regarding the need for standard pharmacy dispensing practices and the application of standardized application of national clinical outcome definitions. An EWI national meeting took place with representatives of all ART sites present. Because data quality assessment proved to be a critical component of EWI monitoring, this meeting was conducted with the goal of training ART sites how to validate their own EWI electronic data with their paper medical records.

EWI data quality assessments revealed differences between pre-validated and validated data due to inconsistencies between record systems and lack of cross-linkages. Data quality assessment is paramount to appropriate reporting and, when routinely practiced, serves to strengthen performance of staff and highlight areas of weakness leading to stronger health care systems. Importantly, validated data gives program managers confidence in the data for use in operations, planning and decision-making. Thus, the results of data quality assessments can help inform changes that will improve patient monitoring systems and clinic practice in order to minimize the emergence of HIVDR.

One important limitation of this exercise is that Namibia could evaluate only three out of the five selected EWIs. Strengthening ART record systems, as mentioned above, will help in alleviating this limitation in future EWI rounds. An additional limitation is that although data for satellite/outreach and IMAI sites were included within the main ART sites, they could not be disaggregated and analyzed separately. Plans are being made to strengthen record keeping systems to allow routine capture of data necessary to disaggregate these sites for analyses. An additional limitation of this exercise is that EWIs were not conducted at private ART sites which limit our ability to make broader statements on ART delivery as a whole in Namibia. Although private ART sites deliver ART at an individual level with regimen selection based on HIVDR testing, programmatic data on these ART-experienced sites would be valuable. Plans are currently being made to pilot EWIs at the private ART sites in Namibia in 2013. An additional limitation of this report is that pediatric patients were not included; however, pediatric EWI monitoring assessing the use and availability of pediatric formulations and weight-based dosing will be implemented.

Despite lessons learned from the EWI pilot in Namibia (2009) [22], ART site performance seem not to have improved in this second round of EWIs (2010); subsequent rounds of EWI monitoring would provide meaningful evaluation in terms of the EWI trends over time. Importantly, results from the first two years of EWI monitoring in Namibia have resulted in public health action, specifically performance feedback to individual ART sites, as well as to the national ART program. These data have resulted in important programmatic changes and operational research which will optimize patient care and minimize preventable HIVDR. Over the next five years, as capacity to monitor EWIs builds throughout Namibia utilizing WHOs updated EWI guidance [17], the MoHSS will expand EWI monitoring to engage IMAI and satellite/outreach sites, the private sector, and pediatric patient populations.
